# Mycobacteriophage maravista: a cluster F1 phage discovered on Cape Cod, Massachusetts

**DOI:** 10.1128/mra.00502-24

**Published:** 2024-06-11

**Authors:** Charles Pelagalli, Debbie-Jacobs Sera, Joseph A. DeGiorgis, Kathleen Cornely

**Affiliations:** 1 Department of Chemistry and Biochemistry, Providence College, Providence, Rhode Island, USA; 2 Department of Biology, University of Pittsburgh, Pittsburgh, Pennsylvania, USA; 3 Department of Biology, Providence College, Providence, Rhode Island, USA; 4 Whitman Center, Marine Biological Laboratory, Woods Hole, Massachusetts, USA; Queens College Department of Biology, Queens, New York, USA

**Keywords:** bacteriophage, mycobacteria, genome analysis

## Abstract

*Mycobacterium virus Maravista*, a member of the family *Gracegardnervirianae* and species *Cheoctovirus*, is an F1 cluster phage that infects *Mycobacterium smegmatis* mc²155. The Maravista genome has 61.3% GC content, is 60,140 bp in length, and encodes 104 putative genes. Maravista encodes two putative glycosyltransferases, suggesting glycosylation of its capsid protein.

## ANNOUNCEMENT

**Fig 1:**
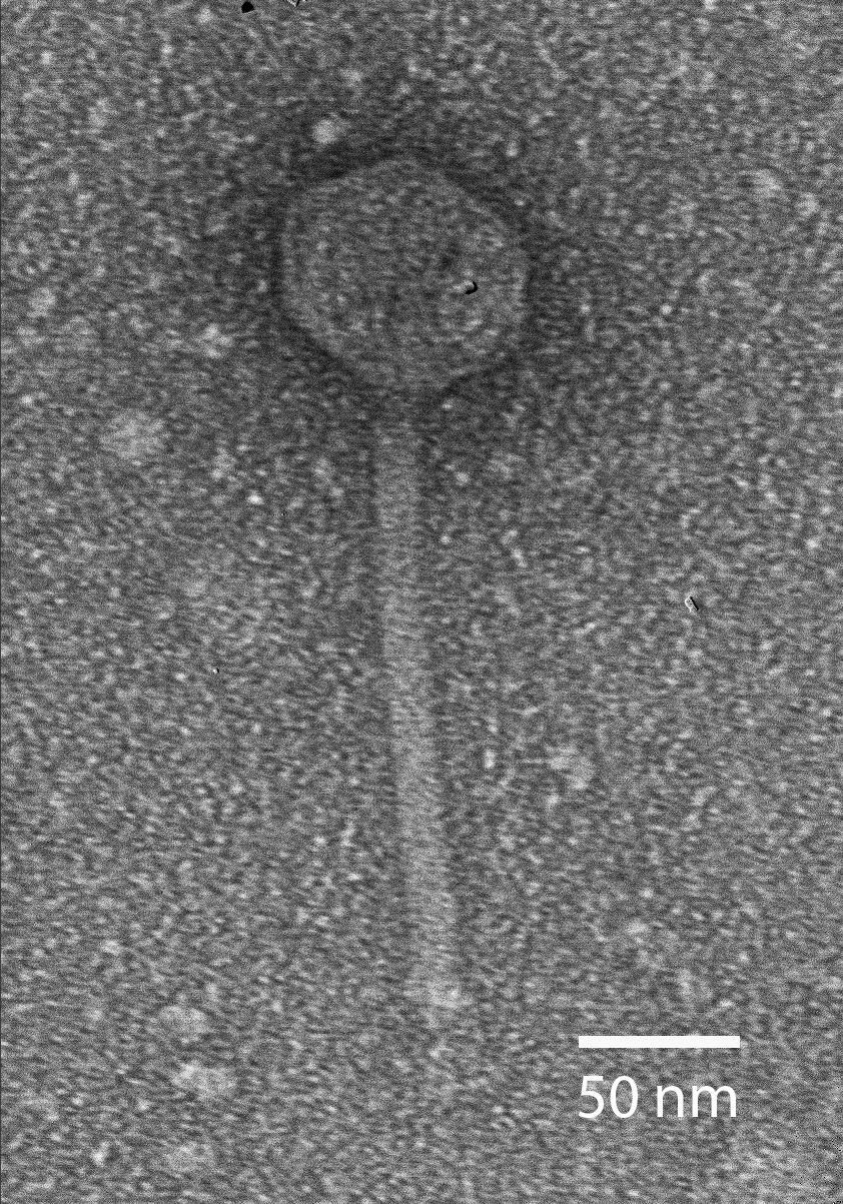
Electron microscopy reveals the siphovirus morphology of Maravista. The image was taken with a JEOL 200 CX transmission electron microscope, using a high-titer lysate negative-stained with 1% uranyl acetate. Maravista features an icosahedral capsid of diameter 67–70 nm and a tail length of 194–197 nm (*n* = 20).

Mycobacteriophages target bacteria of the *Mycobacterium* genus, which includes pathogens responsible for tuberculosis and leprosy (1). Lytic bacteriophages, and temperate bacteriophages genetically engineered to be lytic only, have been used to treat an infection caused by an antibiotic-resistant strain of *Mycobacterium abscessus* (2). Maravista was isolated from a damp soil sample collected under a layer of garden mulch at a residence in Falmouth, Massachusetts (41.548611 N, 70.586111 W) and was purified to homogeneity through four rounds of purification using standard methods (3). A soil sample was treated with 7H9 liquid medium, filtered (0.2 µm pore size), and inoculated with *Mycobacterium smegmatis* mc^2^ 155. The sample was incubated at 37°C with shaking for 48 hours, then plated on top of agar with host bacteria to form plaques. Maravista formed small, clear plaques with a diameter of 1 mm. Negative-stain transmission electron microscopy revealed that Maravista exhibited a siphovirus morphology as shown in Fig. 1.

Phage DNA was isolated from a high titer lysate by phenol:chloroform:isoamyl alcohol extraction (4). The complete genome was sequenced using an Illumina NovaSeq 6000 instrument and a TruSeq DNA Nano Prep kit (5). Consed v29.0 was used to verify the accuracy of raw reads (6), and Newbler v2.9 was used for assembly (7) (Table 1). A total of 42 putative functions were assigned to the 104 protein-coding genes of Maravista’s complete genome sequence. tRNAscan-SE (8) and Aragorn (9) did not identify any tRNA genes in the genome sequence of Maravista. The genome sequence was annotated using DNA Master v5.23.6 (10), while Glimmer v3.02 (11), Starterator v1.1 (12), and GeneMark v2.5 (13) were used to predict the start sites of genes. Functions were assigned to genes using data from HHPred (14), TMHMM v2.0 (15), TOPCONS v2.0 (16), and BLASTp (17). Default parameters were used for all applications. Phamerator v520 (18) was used to compare the sequence similarity of Maravista to closely related F1 subcluster phages; it was determined that Maravista is 98% similar to mycobacteriophageZizzle (MZ274306).

**TABLE 1:** Sequencing, genome, and phage characteristics

Parameter	Phage data
Soil sample characteristics	
Collection date	October 2022
Collection location coordinates	41.548611 N, 70.586111 W
Phage particle characteristics	
Capsid size (nm)	67–70 (*n* = 20)
Tail length (nm)	194–197 (*n* = 20)
Sequencing	
Sequencing instrument	Illumina NovaSeq 6000
Library prep kit	TruSeq DNA Nano Prep
Number of reads	80,000
Length of reads (bp)	150 base single-end reads
Shotgun coverage (×)	233
Phage genome characteristics	
Genome length (bp)	60,140
3’ single-stranded overhang	10 bases (5′-CGGAAGGCGC-3′)
GC content (%)	61.3%
attP site (bp)	32,545–32,566

Only three genes were identified as belonging to orphans. Structural genes on the left arm of the genome are typical of related F1 subcluster phages, encoding terminase large and small subunits, a portal protein, a capsid maturation protease, scaffolding protein, head-to-tail adaptors and stoppers, and tail assembly chaperones, the latter exhibiting a +1 translational frameshift. Maravista encodes putative genes involved in lysogeny—a tyrosine integrase, an immunity repressor, and an excise enzyme—and is thus likely to be able to adopt a temperate lifestyle.

Maravista encodes multiple putative glycosyltransferases near the 3’ end of its genome, which suggests that the phage’s major capsid proteins have the potential to be glycosylated. Bacteriophages are not typically glycosylated, as they do not enter their hosts as eukaryotic viruses do; however, recently, it has been reported that closely related F1 cluster phages Che8 (19) and Rita (20, 21) have glycan groups covalently attached to major capsid proteins. This results in the formation of a glycan shield, which can alter antibody production and prevent binding of immunoglobins to the capsid which may provide an advantage in phage therapy (19).

## Data Availability

Maravista is available at GenBank under accession number PP208938. The sequence read archive (SRA) number is SRX23702565.

## References

[1] Hatfull GF. 2012. Chapter 7 - the secret lives of mycobacteriophages, p 179–288. In Łobock M, Szybalski WT (ed), Advances in virus research. Academic Press. 10.1016/B978-0-12-394621-8.00015-7 22420855

[2] Dedrick RM, Guerrero-Bustamante CA, Garlena RA, Russell DA, Ford K, Harris K, Gilmour KC, Soothill J, Jacobs-Sera D, Schooley RT, Hatfull GF, Spencer H. 2019. Engineered bacteriophages for treatment of a patient with a disseminated drug-resistant Mycobacterium abscessus. Nat Med 25:730–733. doi:10.1038/s41591-019-0437-z 31068712 PMC6557439

[3] Poxleitner: phage discovery guide. 2024. Google scholar. Available from: https://scholar.google.com/scholar_lookup?title=Phage+discovery+guide&publication_year=2018. Retrieved 06 May 2024.

[4] Spada S. 2020. Chapter 6 - methods to purify DNA from extracellular vesicles: focus on exosomes, p 109–118. In Spada S, Galluzzi L (ed), Methods in enzymology. Academic Press. 10.1016/bs.mie.2020.09.004 33565966

[5] Russell DA. 2018. Sequencing, assembling, and finishing complete bacteriophage genomes, p 109–125. In Clokie MRJ, Kropinski AM, Lavigne R (ed), Bacteriophages: methods and protocols. Springer, New York, NY. 10.1007/978-1-4939-7343-9_9 29134591

[6] Gordon D, Green P. 2013. Consed: a graphical editor for next-generation sequencing. Bioinformatics 29:2936–2937. doi:10.1093/bioinformatics/btt515 23995391 PMC3810858

[7] Margulies M, Egholm M, Altman WE, Attiya S, Bader JS, Bemben LA, Berka J, Braverman MS, Chen Y-J, Chen Z, et al.. 2005. Genome sequencing in microfabricated high-density picolitre reactors. Nature 437:376–380. doi:10.1038/nature03959 16056220 PMC1464427

[8] Lowe TM, Eddy SR. 1997. tRNAscan-SE: a program for improved detection of transfer RNA genes in genomic sequence. Nucleic Acids Res 25:955–964. doi:10.1093/nar/25.5.955 9023104 PMC146525

[9] Laslett D, Canback B. 2004. ARAGORN, a program to detect tRNA genes and tmRNA genes in nucleotide sequences. Nucleic Acids Res 32:11–16. doi:10.1093/nar/gkh152 14704338 PMC373265

[10] Pope WH, Jacobs-Sera D. 2018. Annotation of bacteriophage genome sequences using DNA master: an overview, p 217–229. In Clokie MRJ, Kropinski AM, Lavigne R (ed), Bacteriophages: methods and protocols. Springer, New York, NY. 10.1007/978-1-4939-7343-9_16 29134598

[11] Delcher AL, Harmon D, Kasif S, White O, Salzberg SL. 1999. Improved microbial gene identification with GLIMMER. Nucleic Acids Res 27:4636–4641. doi:10.1093/nar/27.23.4636 10556321 PMC148753

[12] Pacey M. 2016. Starterator guide. Pittsburgh, PA University of Pittsburgh

[13] Lukashin AV, Borodovsky M. 1998. GeneMark.hmm: new solutions for gene finding. Nucleic Acids Res 26:1107–1115. doi:10.1093/nar/26.4.1107 9461475 PMC147337

[14] Söding J, Biegert A, Lupas AN. 2005. The HHpred interactive server for protein homology detection and structure prediction. Nucleic Acids Res 33:W244–W248. doi:10.1093/nar/gki408 15980461 PMC1160169

[15] Möller S, Croning MDR, Apweiler R. 2001. Evaluation of methods for the prediction of membrane spanning regions. Bioinformatics 17:646–653. doi:10.1093/bioinformatics/17.7.646 11448883

[16] Tsirigos KD, Peters C, Shu N, Käll L, Elofsson A. 2015. The TOPCONS web server for consensus prediction of membrane protein topology and signal peptides. Nucleic Acids Res 43:W401–W407. doi:10.1093/nar/gkv485 25969446 PMC4489233

[17] Altschul SF, Gish W, Miller W, Myers EW, Lipman DJ. 1990. Basic local alignment search tool. J Mol Biol 215:403–410. doi:10.1016/S0022-2836(05)80360-2 2231712

[18] Cresawn SG, Bogel M, Day N, Jacobs-Sera D, Hendrix RW, Hatfull GF. 2011. Phamerator: a bioinformatic tool for comparative bacteriophage genomics. BMC Bioinformatics 12:395. doi:10.1186/1471-2105-12-395 21991981 PMC3233612

[19] Freeman KG, Robotham AC, Parks OB, Abad L, Jacobs-Sera D, Lauer MJ, Podgorski JM, Zhang Y, Williams JV, White SJ, Kelly JF, Hatfull GF, Pope WH. 2023. Virion glycosylation influences mycobacteriophage immune recognition. Cell Host Microbe 31:1216–1231. doi:10.1016/j.chom.2023.05.028 37329881 PMC10527164

[20] Fakhri AM, Warner MH, DeGiorgis JA, Cornely K. 2023. Mycobacteriophage Rita: a cluster F1 phage discovered in North Easton, Massachusetts. Microbiol Resour Announc 12:e0051023. doi:10.1128/MRA.00510-23 37638726 PMC10508093

[21] View - poster. Scimeetings | ACS. Available from: https://scimeetings.acs.org/exhibit/analysis-discovery-mycobatceriophage-Rita/3997135. Retrieved 08 May 2024.

[22] Jordan TC, Burnett SH, Carson S, Caruso SM, Clase K, DeJong RJ, Dennehy JJ, Denver DR, Dunbar D, Elgin SCR, et al.. 2014. A broadly implementable research course in phage discovery and genomics for first-year undergraduate students. mBio 5:e01051-13. doi:10.1128/mBio.01051-13 24496795 PMC3950523

